# Supplementary material for: Comprehensive Review: Unveiling the Pro-Oncogenic Roles of IL-1ß and PD-1/PD-L1 in NSCLC Development and Targeting Their Pathways for Clinical Management
Source: Int J Mol Sci. 2023 Jul 17;24(14):11547. doi: 10.3390/ijms241411547 (PMC10380530; doi:10.3390/ijms241411547)
Supplement: Supplementary file 1 [file ijms-24-11547-s001.zip › ijms-2497376-supplementary.pdf]

Table S1. List of PD-1/PD-L1 inhibitors and corresponding clinical trials for NSCLC.

**Name**	**Class**	**Target**	**Phase**	**n =**	**Primary Outcome**	**Control vs. intervention arms**
Pembrolizumab	IgG4 engineered humanized antibody	PD-1	NCT01905657 (phase II/III) (KEYNOTE-010)	1034	OS, PFS, AEs, ORR	Pembrolizumab 2 mg/kg versus Pembrolizumab 10 mg/kg versus Docetaxel
			NCT02142738 (phase III) (KEYNOTE-024)	305	PFS	Pembrolizumab versus SOC chemotherapy
Nivolumab	IgG4 fully human antibody	PD-1	NCT01673867 (phase III) (CheckMate057)	792	OS, one year OS, number of deaths	Nivolumab versus Docetaxel
			NCT01642004 (phase III) (CheckMate 017)	352	OS, number of deaths	Nivolumab versus Docetaxel
			NCT02041533 (phase III) (CheckMate 026)	1325	PFS	Nivolumab versus Investigator choice of chemotherapy
			NCT02713867 (phase III) (CheckMate 384)	363	PFSR by 6 months and 12 months	Nivolumab 480 mg Q4W versus Nivolumab 240 mg Q2W
Cemiplimab	fully humanized IgG4 mAb	PD-1	NCT03409614 (phase III) (EMPOWER-Lung 3)	Active recruitment	OS	Platinum-based doublet chemotherapy versus Cemiplimab + Platinum-based doublet chemotherapy
			NCT03515629 (phase III)	Terminated	PFS	Pembrolizumab versus Cemiplimab + ipilimumab versus Cemiplimab plus chemotherapy + ipilimumab
			NCT03088540 (phase III) (EMPOWER-Lung 1)	712	OS, PFS	Standard-of-care chemotherapy versus Cemiplimab
Durvalumab	human IgG1 kappa	PD-L1	NCT02125461 (phase III) (PACIFIC)	713	PFS, OS	Durvalumab versus Placebo
			NCT02453282 (phase III) (MYSTIC)	1118	OS, PFS	Durvalumab Monotherapy versus Durvalumab + Tremelimumab versus SoC chemotherapy

Atezolizumab	IgG1 monoclonal antibody	PD-L1	NCT02367794 (phase III) (IMpower131)	1021	PFS, OS	Nab-Paclitaxel + Carboplatin versus Atezolizumab + Nab-Paclitaxel + Carboplatin versus Atezolizumab + Paclitaxel + Carboplatin
			NCT02409342 (phase III) (IMpower110)	572	OS	Chemotherapy (Carboplatin/ Cisplatin) + (Pemetrexed/ Gemcitabine) versus Atezolizumab
			NCT02008227 (phase III) (OAK)	1225	% of patients who died, OS	Docetaxel versus Atezolizumab
			NCT02367781 (phase III) (IMpower130)	723	PFS, OS	Atezolizumab + Nab-Paclitaxel + Carboplatin versus Nab-Paclitaxel + Carboplatin
			NCT02366143 (phase III) (IMpower150)	1202	PFS, OS	Bevacizumab + Paclitaxel + Carboplatin versus Atezolizumab + Bevacizumab + Paclitaxel + Carboplatin versus Atezolizumab + Paclitaxel + Carboplatin
Avelumab	completely human IgG1 monoclonal antibody	PD-L1	NCT02395172 (phase III) (JAVELIN Lung 200)	792	OS	Avelumab versus Docetaxel

Legend: PD-1—programmed cell death 1; PD-L1—programmed death ligand 1; OS—overall survival; PFS—progression-free survival; AE—adverse events; PFSR—progression-free survival rate

Table S2. Immune-Related Adverse Events (irAEs) related to PD-1/PD-L1 inhibitors.

**Drug name**	**Mechanisms**	**More common irAEs [104–106]**	**Adverse reaction rate (Grade 3-5) [107]**
Pembrolizumab	PD-1 inhibitors	Rash, pruritus, diarrhea, transaminitis, hypothyroidism	16.58%
Nivolumab	PD-1 inhibitors	Rash, pruritus, Vitiligo, diarrhea	15.72%
Cemiplimab	PD-1 inhibitors	Diarrhea, transaminitis	18% [108]

Legend: Adverse events caused by immunotherapy were termed immune-related adverse events (irAEs), with an incidence between 15 and 90% [104]. Compared to PD-1 inhibitors, grade 3–4 irAEs were more common with anti-CTLA-4 agents (31% vs.10%) [109]. Pneumonitis, hypothyroidism, arthralgias,

and vitiligo are more common in patients treated with anti-PD-1 inhibitors [107].
